# Supplementary material for: Interplay between p300 and HDAC1 regulate acetylation and stability of Api5 to regulate cell proliferation
Source: Sci Rep. 2021 Aug 12;11:16427. doi: 10.1038/s41598-021-95941-4 (PMC8361156; doi:10.1038/s41598-021-95941-4)
Supplement: Supplementary file 1 — Supplementary Information 1. [file 41598_2021_95941_MOESM1_ESM.pdf]

Supplementary Materials

Interplay between p300 and HDAC1 regulate acetylation and stability of Api5 to regulate cell proliferation

Virender Kumar Sharma and Mayurika Lahiri*

Department of Biology, Indian Institute of Science Education and Research, Dr. Homi Bhabha Road, Pune, Maharashtra 411008, India

*Author for correspondence (mayurika.lahiri@iiserpune.ac.in)

Supplementary Figure S1

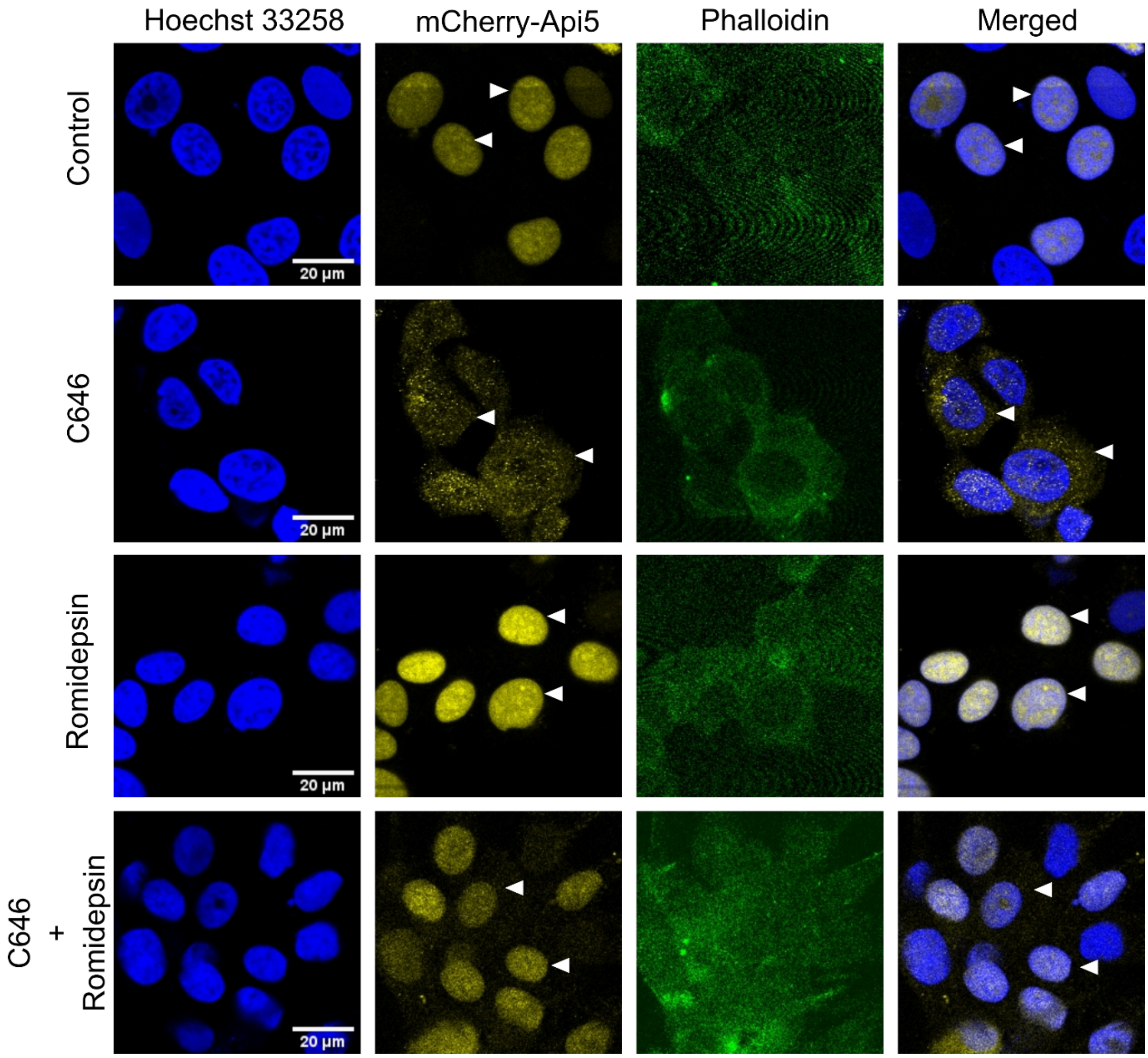

Supplementary Figure S1: p300 and HDAC1 regulate Api5 localisation

mCherry tagged Api5 over-expressing MCF7 cells were treated with 7.5 µM C646, 0.1 nM romidepsin for 4 hrs and 16 hrs respectively. mCherry fluorescence was visualised using confocal microscopy (scale bar: 20 µm).

Supplementary Figure S2

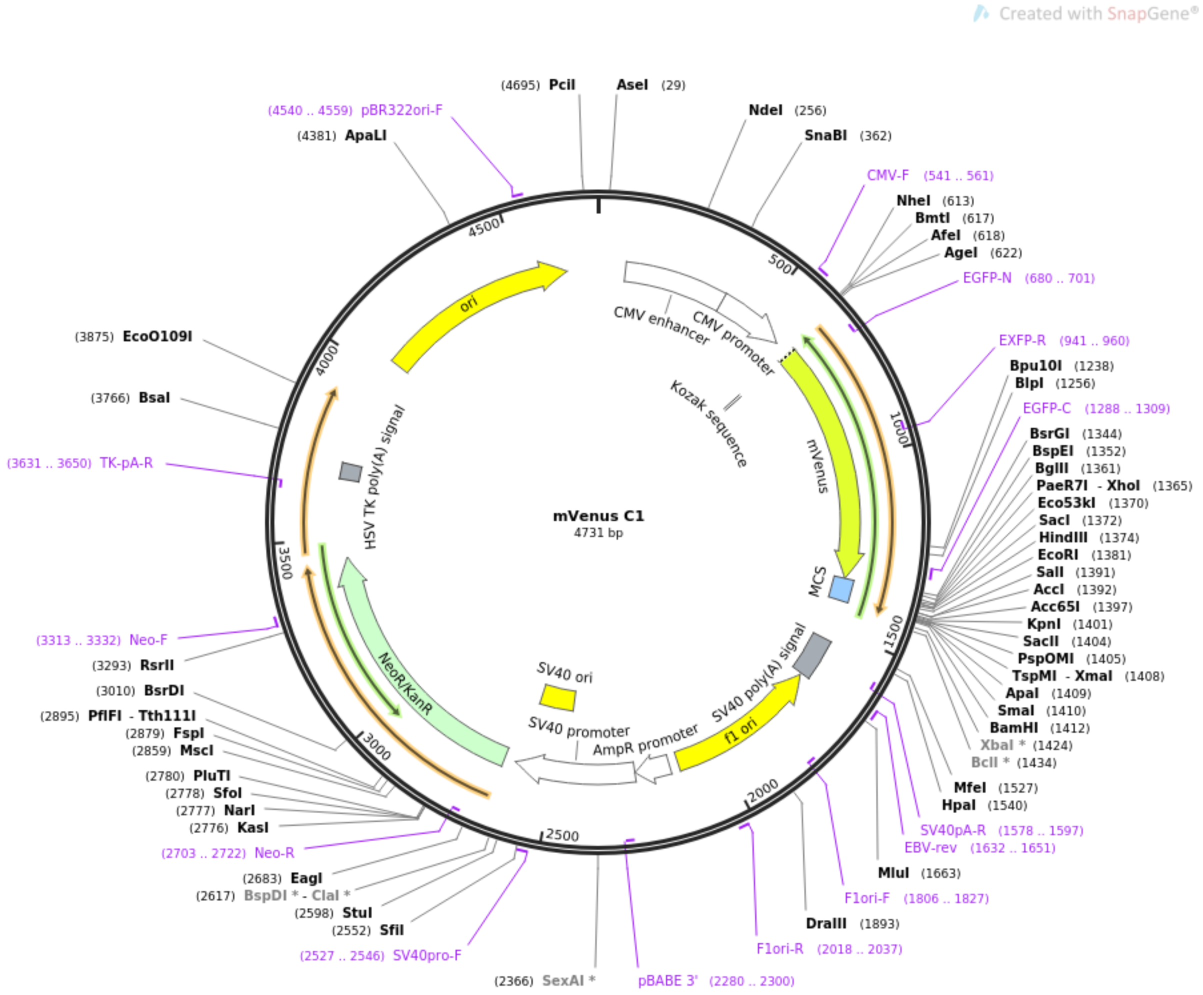

mVenusC1 was gifted by Jennifer Lippincott-Schwartz, NIH, USA in which Api5 was cloned. The vector map was obtained from Addgene web site.

Supplementary Figure S3

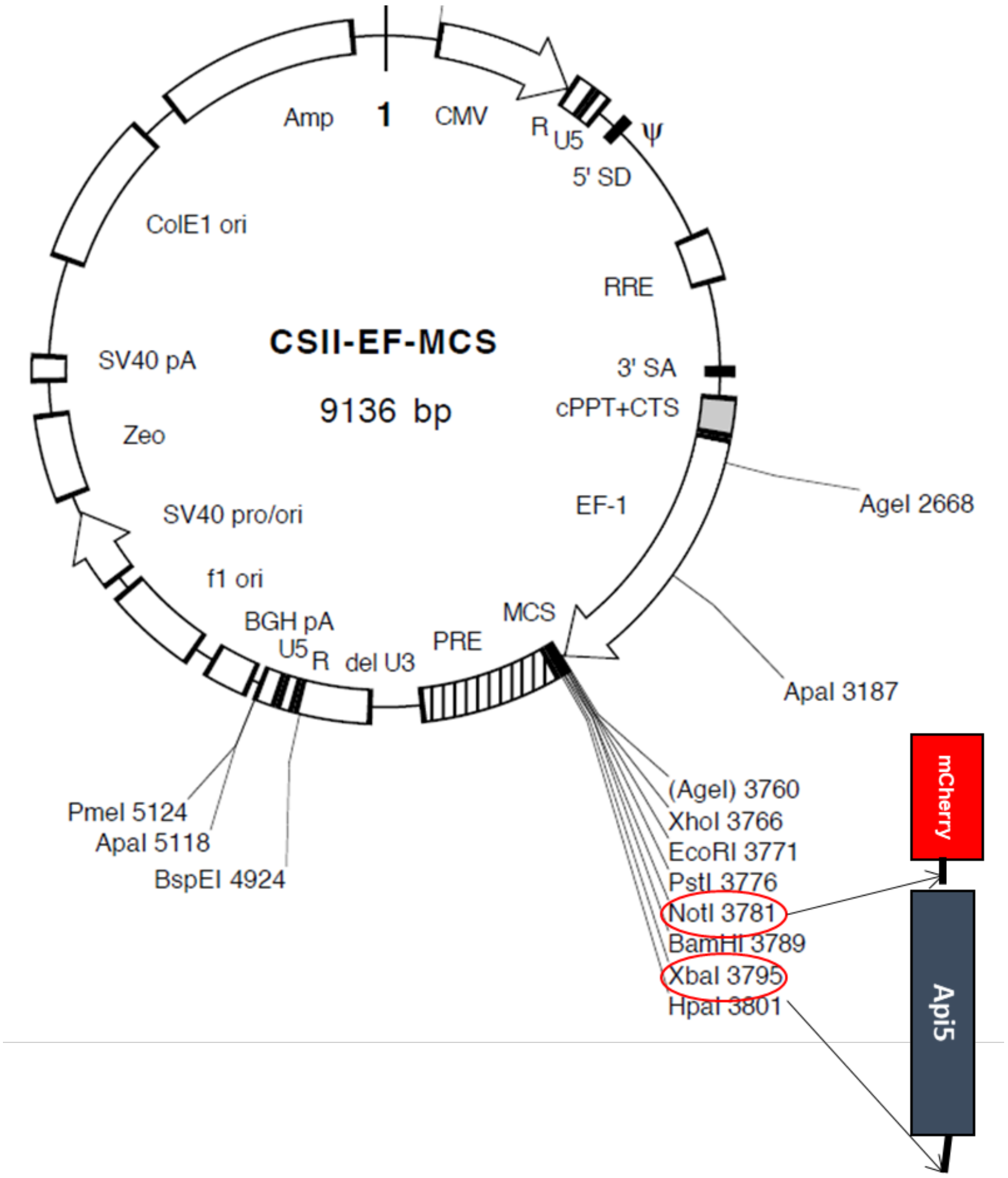

CSII-EF-MCS plasmid was a gift from Dr. Sourav Banerjee, NBRC, Manesar, India. Api5 was cloned into CSII-EF-MCS vector with a mCherry tag between NotI and XbaI sites. The vector map of CSII-EF-MCS plasmid was provided by Riken BioResource Centre, Japan.

Supplementary Figure S4

a.

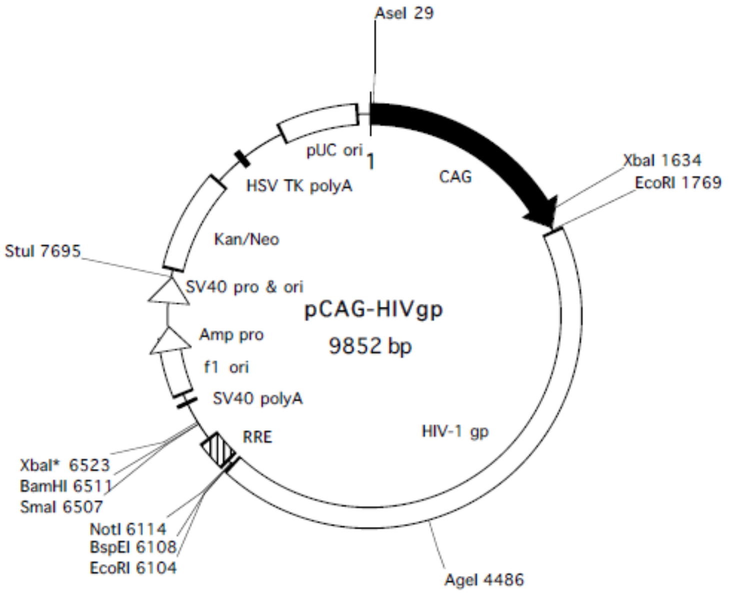

b.

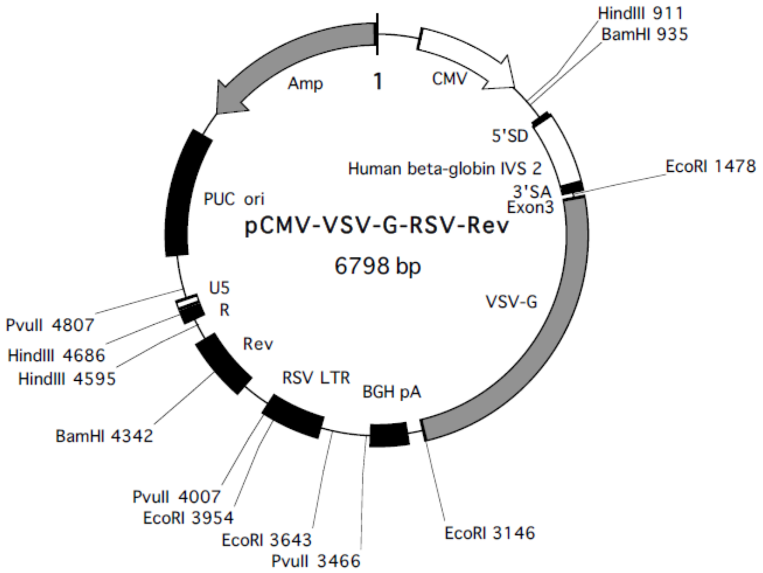

a) Plasmid details of expression vector of HIV-1 gag and pol for lentivirus vector packaging and b) envelope VSVG protein and Rev for producing lentiviral particles to generate mCherry-Api5 stable cell lines. The plasmids was purchased from RIKEN BioResource Centre, Japan and the vector map was provided by them.

Supplementary Figure S5: Full blots for Figures 2a and 3a.

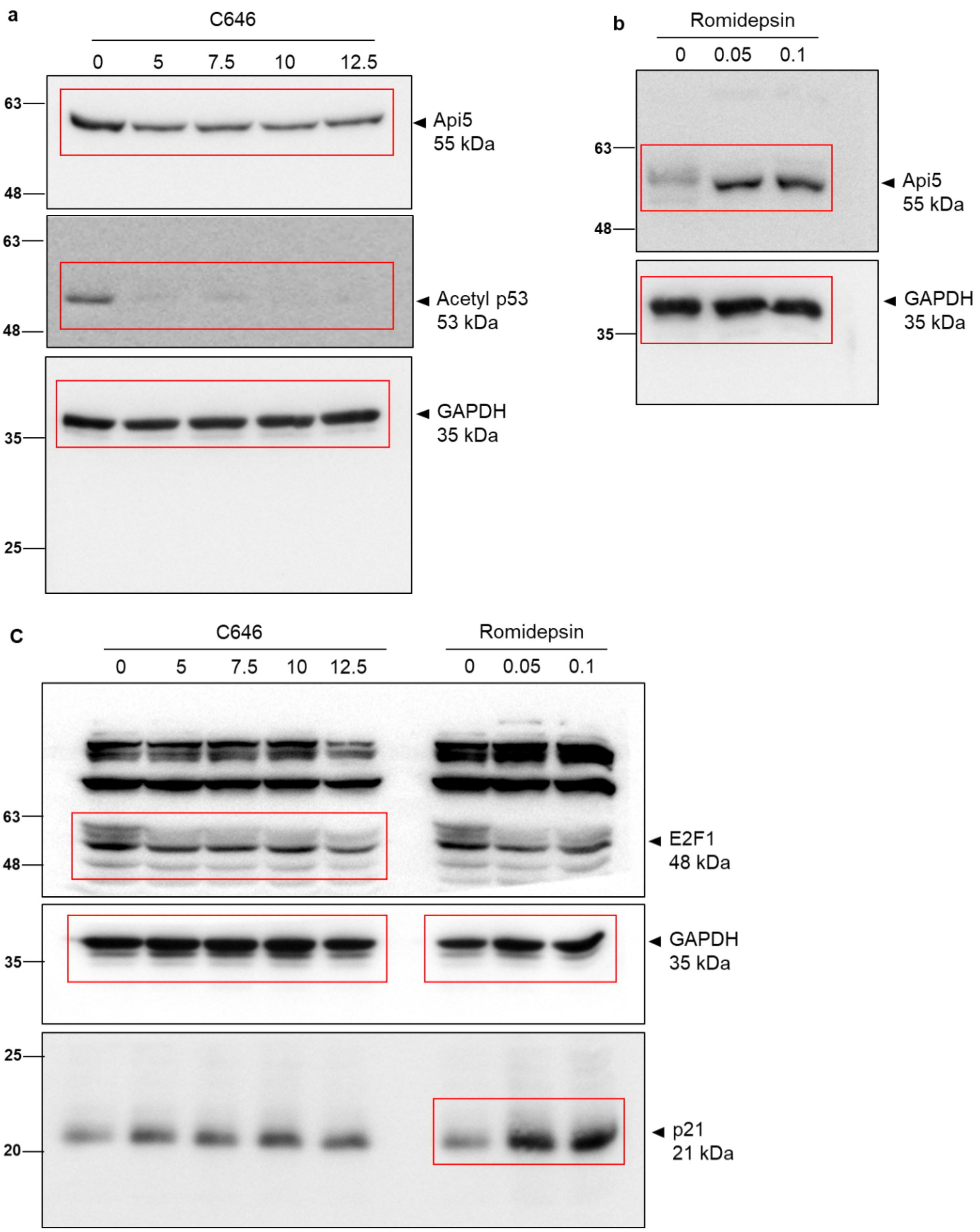

Supplementary Figure S6: Agarose gels for Figures 2e and 3d

a

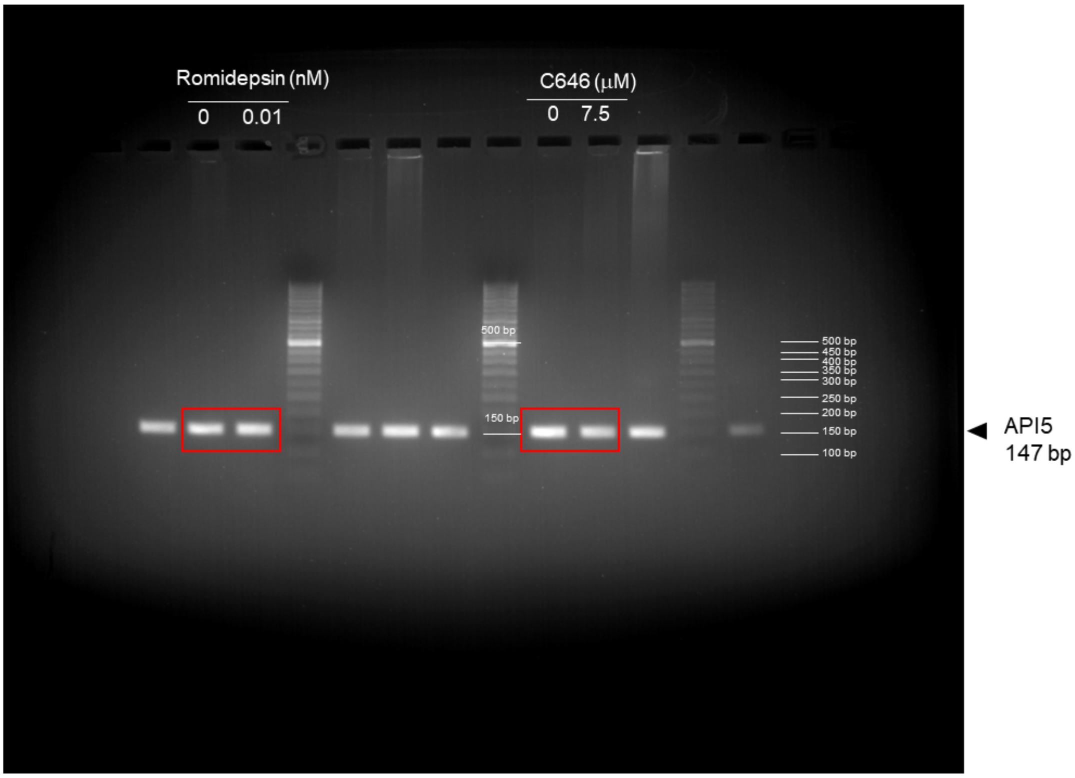

b

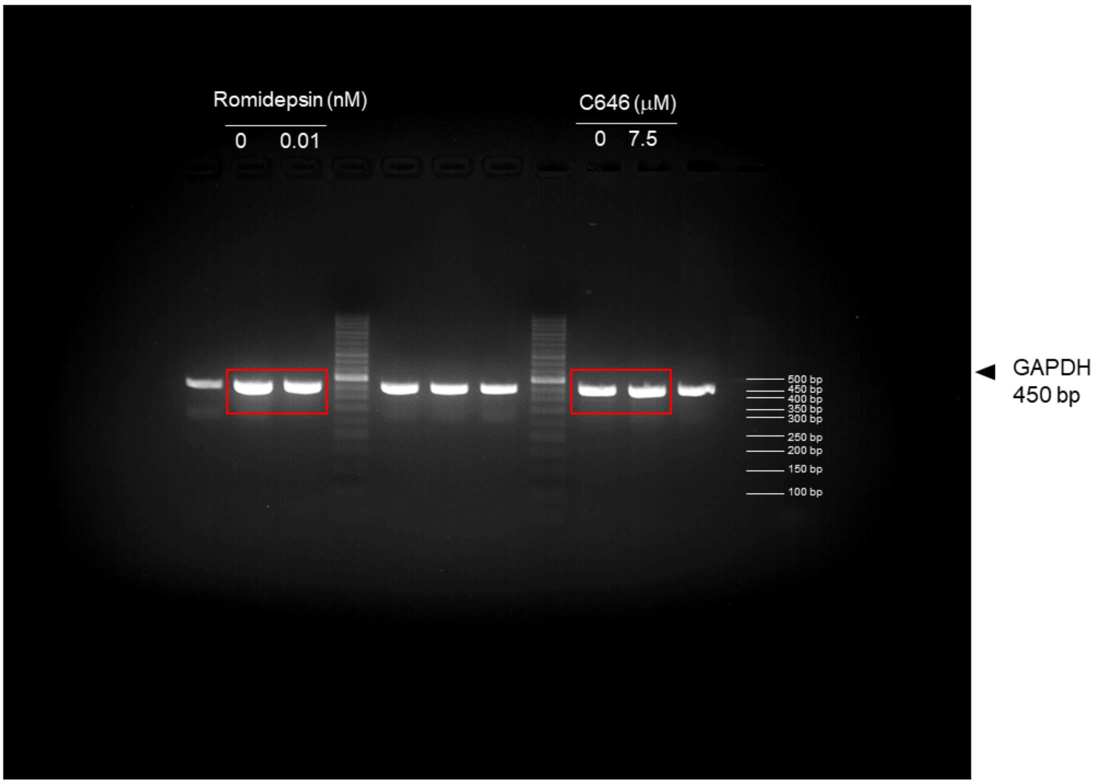

c

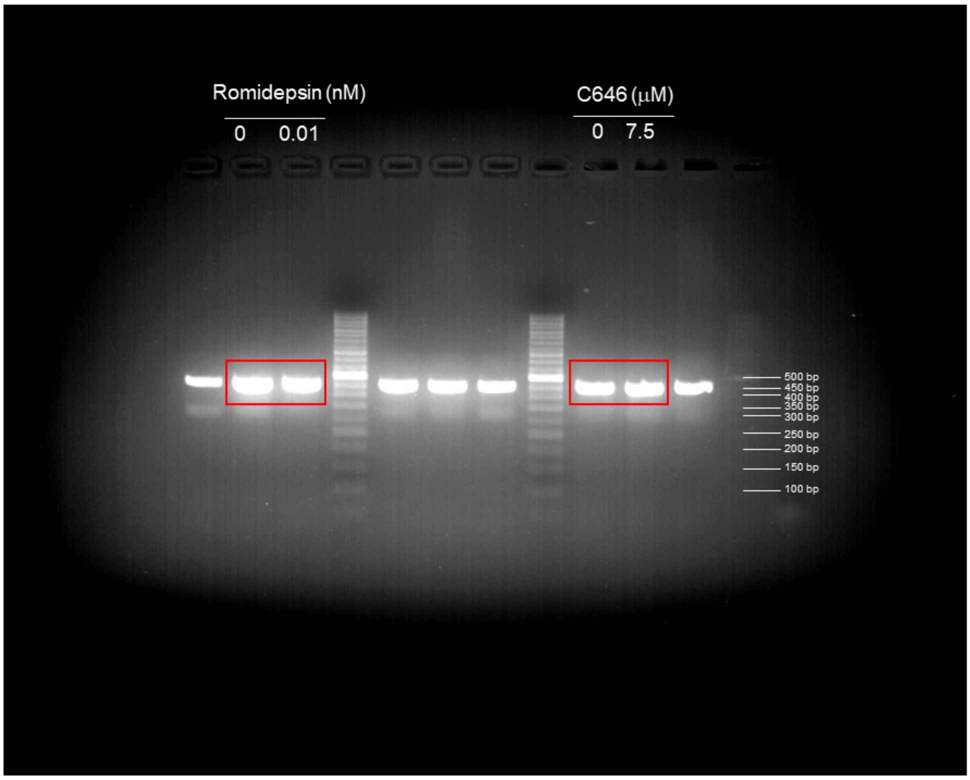

The PCR was set up as mentioned in materials and methods. We prepared cDNA master mix and aliquoted 10 μL of reaction mixture into separate tubes and added the API5 and GAPDH primers respectively. The PCR reaction was carried out in the same PCR machine with gradient temperature. The electrophoresis of PCR products was carried out in two separate gels for a) API5 and b) GAPDH low exposure image and c) GAPDH high exposure image.

Supplementary Figure S7: Full blots for Figure 2g and 2h.

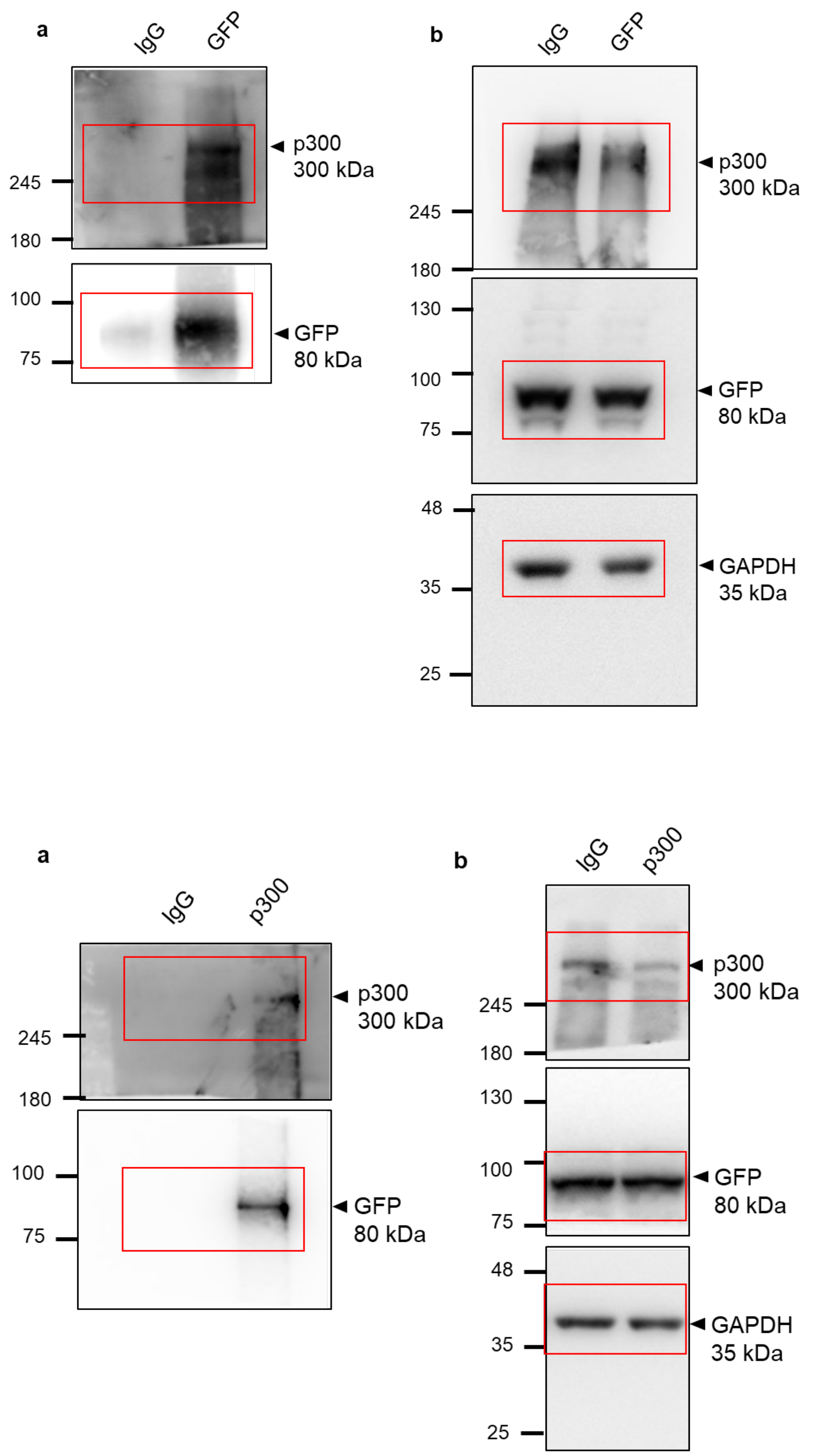

Supplementary Figure S8: Full blots for Figure 3f and 3g.

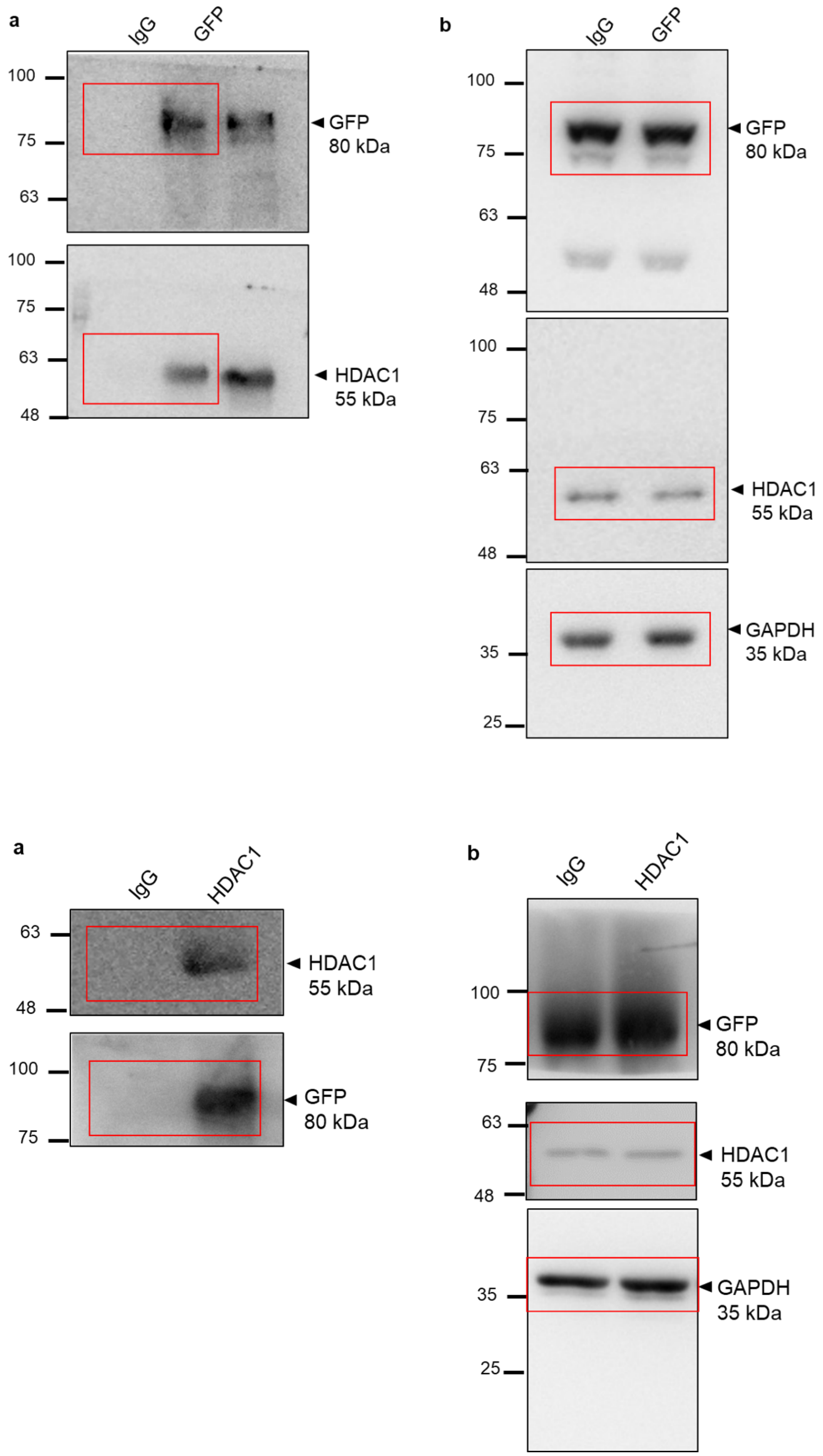

Supplementary Figure S9: Full blots for Figure 4a and b.

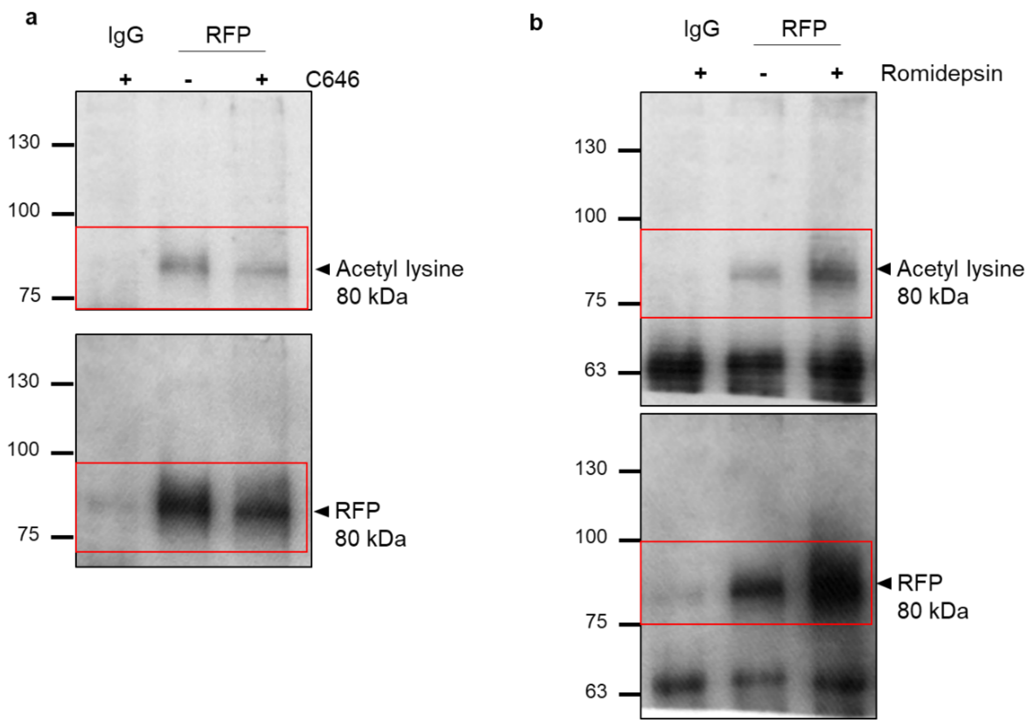

Supplementary Figure S10: Full blots for Figure 4c and e.

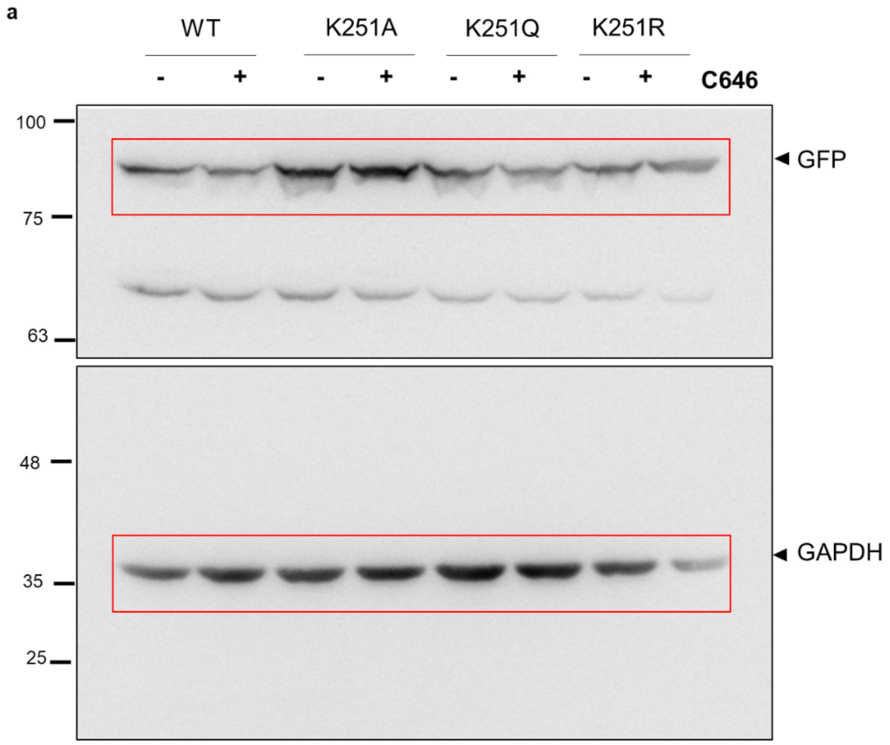

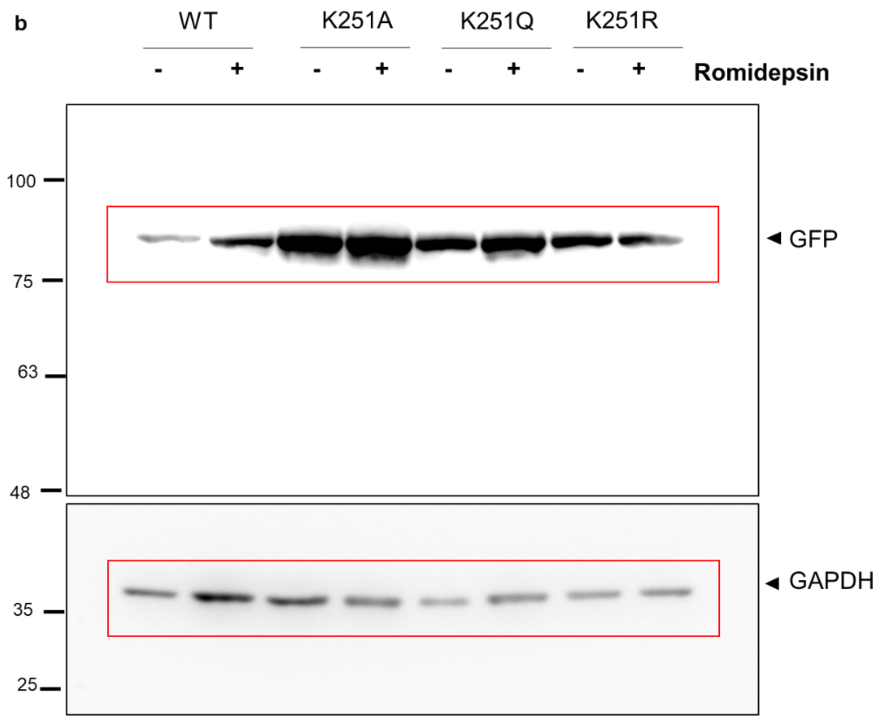

Supplementary Figure S11: Full blots for Figure 6b.

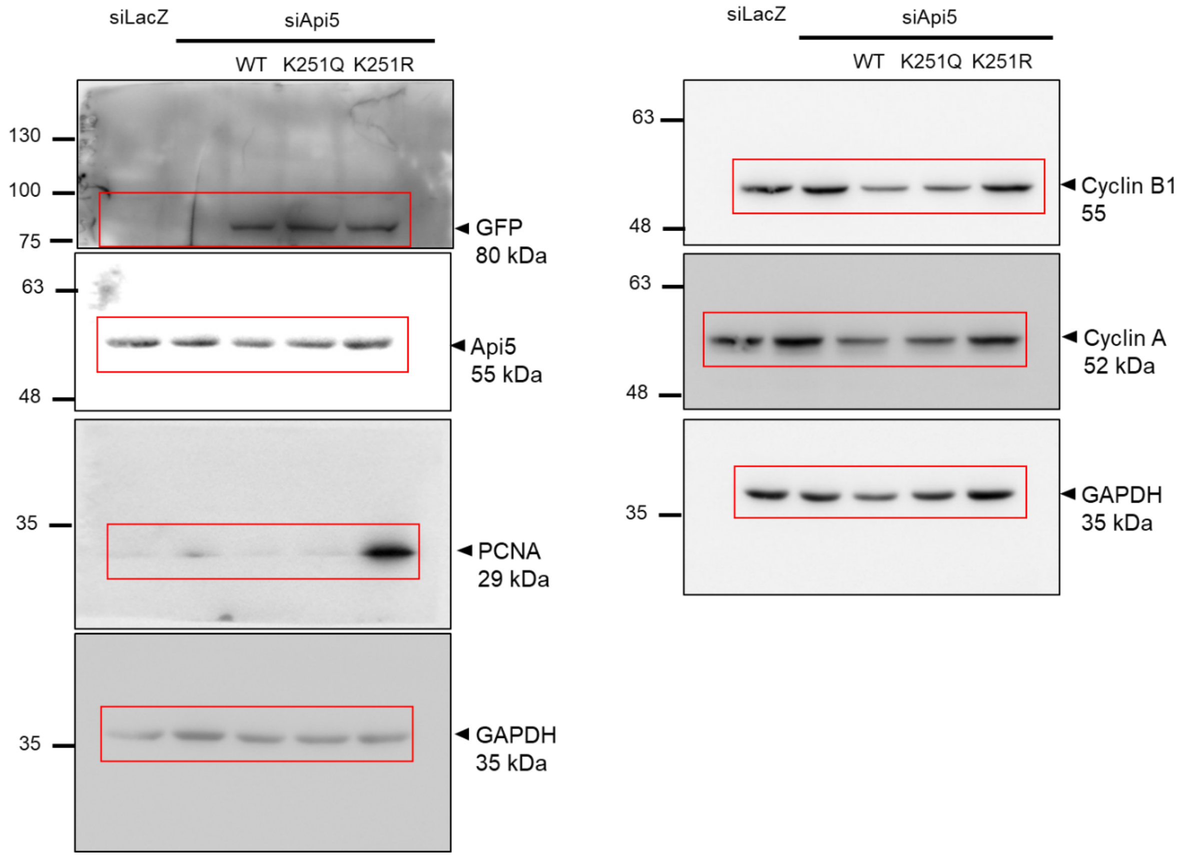

Supplementary Figure S12: Full blots for Figure 7.

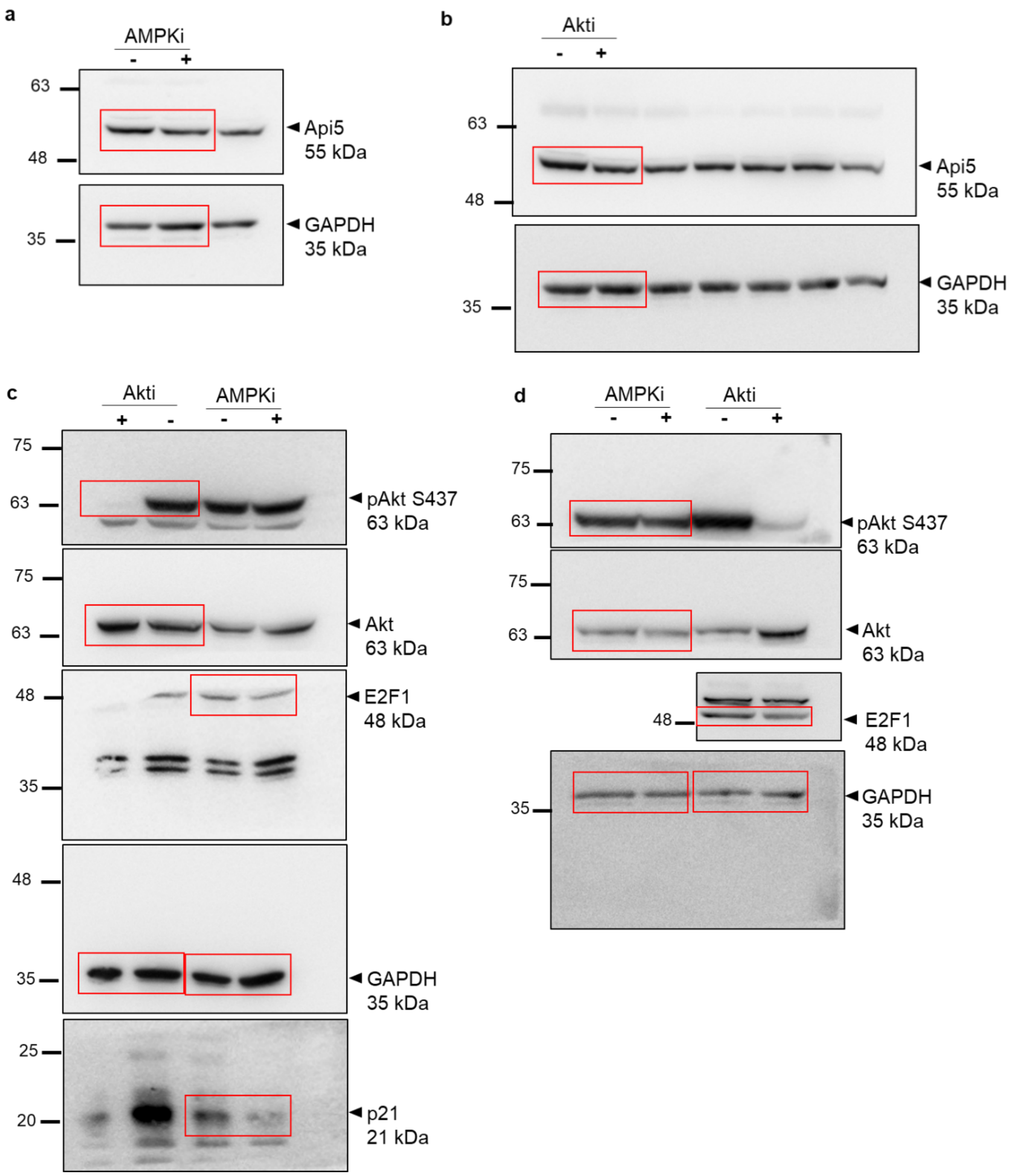

Supplementary information

Primers used for cloning of Api5 into mVenus C1 and mCherry CSII-EF MCS vectors

mVenus Api5 For: GGAAGATCTATGCCGACAGTAGAGGAGCT

mVenus Api5 Rev: CCGGAATTCTCAGTAGAGTCTTCCCCGAC

mCherry Api5 For: AAGGAAAAAAGCGGCCGCATATGCCGACAGTAGAGGAGCT

mCherry Api5 Rev : GCTCTAGATCAGTAGAGTCTTCCCCGAC

Cloning of Api5 into mVenus C1 or mCherry MCS-CSII-EF

Full length Api5 was cloned into mVenus C1 vector after amplifying it using mVenus API5 forward and reverse primers. This wild type Api5 in mVenus C1 was further cloned into mCherry-CSII-EF-MCS vector for preparing lentiviral plasmids. Api5-mVenus C1 plasmid was PCR amplified (Supplementary Table S1) using mCherry Api5 forward and reverse primers with XbaI and NotI restriction site overhangs. PCR cycle was 95˚C for 60 sec, 60˚C for 1 min, 72˚C for 3 mins sec and final extension for 5 min, repeated for 30 cycles. PCR products were purified using a PCR purification kit (Qiagen, USA) and digested using XbaI (New England Biolabs; NEB USA) and NotI HF (NEB) for 4 hours (Supplementary Table S2). mCherry-CSII-EF-MCS vector was also digested using same restriction enzymes at 37ºC for 3 hours and calf intestinal phosphatase (CIP; NEB) treatment for 1 hr was performed. Digested plasmids were gel extracted after resolving on agarose gel using gel extraction kit (Sigma-Aldrich, USA). Api5 was ligated into mCherry-CSII-EF-MCS vector with an insert to vector ratio of 75 ng :25 ng using T4 DNA ligase (Takara Bio; USA) at 16ºC overnight (Supplementary Table S3). Ligation mixture was used to transform DH5α competent cells and incubated overnight at 37^{0}C. Plasmid DNA was isolated from the obtained colonies and screened by PCR to check for the correct insert.

Supplementary Table S1: PCR amplification of wild type from API5-mVenus C1
Table contains the details of reagents used for PCR amplification of API5 from mVenus C1 for cloning into CSII-EF-MCS vector.

DNA Template	3 μl
Forward Primer	3 μl
Reverse Primer	3 μl
10 mM dNTPs	12.5 μl
10X Pfu buffer	10 μl
DMSO	5 μl
dH_2O	6.5 μl
Pfu polymerase	2 μl

Supplementary Table S2: Digestion of PCR product and CSII-EF-MCS-mCherry
Table contains the details of reagents used for digestion of PCR amplified API5 with XbaI and NotI over hangs and CSII-EF-MCS vector with mCherry tag.

DNA Template	5 μg
10 X Cut smart buffer	5 μl
Not 1 HF	5 μl
Xba 1	5 μl
dH_2O	To make up the reaction to 50 μl

Supplementary Table S3: Ligation reaction of API5 into mCherry tagged CS-II-EF-MCS digested vector
Table contains the details of reagents used for ligating gel-extracted digested products of Api5 and CSII-EF-MCS-mCherry vector.

Insert	75 ng
Vector	25 ng
10X T4 Ligase buffer	1 μl
T4 DNA Ligase	1 μl
dH_2O	To make up the reaction to 10 μl

Site-directed mutagenesis to generate siRNA resistant WT Api5 and K251 Api5 mutants.

The primers for site directed mutagenesis to generate siRNA resistant Api5 and Api5 K251 mutations were designed and ordered from Sigma-Aldrich, USA.

mut Api5 For: 5'-GCCGAACTGGATCAGACCTTCA-3'
mut Api5 1-Rev: 5'-TGAAGGTCTGATCCACTTCGGC-3'
Api5K251Qfor: 5'-CAGTACCCCTCTTCTCTCAGAATGTCCATTCCACAAGGTT-3'
Api5K251Qrev: 5'-AACCTTGTGGAATGGACATTCTGAGAGAAGAGGGGTACTG-3'
Api5K251Rfor: 5'-CAGTACCCCTCTTCTCTCGAAATGTCCATTCCACAAGGTT-3'
Api5K251Rrev: 5'-AACCTTGTGGAATGGACATTTCGAGAGAAGAGGGGTACTG-3'
Api5K251Afor: 5'-CAGTACCCCTCTTCTCTGCAAATGTCCATTCCACAAGGTT-3'
Api5K251Arev: 5'-AACCTTGTGGAATGGACATTTGCAGAGAAGAGGGGTACTG-3'

PCR reaction was set up with WT Api5 mVenus C1 as template with mut Api5 forward and reverse primers to generate siRNA resistant Api5 (Supplementary Table S4). The PCR cycle was as follows: 95˚C for 60 secs, 60˚C for 1 min, 68˚C for 20 mins and final extension for 5 min. The PCR product was DpnI digested at 37^0C for 3 hrs and transformed into DH5α cells. Colonies obtained were screened by sequencing. The siRNA resistant WT Api5 mVenus C1 plasmid was used as a template to generate K251 mutants of Api5 using specific primers and the same procedure.

Supplementary Table S4: PCR amplification cycle to generate mutants of Api5. Table contains the details of reagents used for PCR amplification of API5-mVenus to generate siRNA resistant Api5 and K251 mutants of Api5.

DNA Template	500 ng
Forward Primer (25 μM)	3 μl
Reverse Primer (25 μM)	3 μl
10 mM dNTPs	12.5 μl
10X Pfu turbo buffer	10 μl
DMSO	5 μl
dH_2O	6.5 μl
Pfu turbo polymerase	2 μl
